# Function of molecular-weight-optimized Astragalus polysaccharides in cisplatin-caused acute kidney injury: mechanisms centered on gut microbiota regulation and precise treatment approaches

**DOI:** 10.3389/fmicb.2026.1854645

**Published:** 2026-05-29

**Authors:** Huijing Li, Huiqiong Li, Rongjing Wu, Miao Zhong

**Affiliations:** Nephrology Department, Hunan Aerospace Hospital, The Affiliated Aerospace Hospital of Hunan Normal University, Changsha, China

**Keywords:** 16S rRNA sequencing, acute kidney injury, Astragalus polysaccharides, cisplatin, delivery systems, gut microbiota, gut–kidney axis, molecular weight

## Abstract

Cisplatin is a widely used chemotherapeutic drug for solid tumors, including colorectal cancer, but its clinical application is limited by dose-dependent nephrotoxicity, often resulting in acute kidney injury (AKI). The gut–kidney axis has emerged as a key factor in cisplatin-induced AKI, with gut microbial imbalance contributing to inflammation and metabolic dysregulation. Astragalus polysaccharides (APS), the main bioactive constituents of *Astragalus membranaceus*, have shown potential in mitigating AKI, partly through modulation of the gut microbiota. Clinical sequencing data indicate that cisplatin treatment reduces short-chain fatty acid (SCFA)-producing bacteria (e.g., *Faecalibacterium, Roseburia*) and increases potentially pathogenic groups (e.g., *Enterobacteriaceae*), leading to alterations in SCFA, amino acid, and bile acid metabolism. This study integrates these findings with existing literature to propose a molecular-weight (Mw)-defined APS model targeting the gut–kidney axis. While high-Mw APS (>100 kDa) primarily act via microbial fermentation to restore SCFA production and gut barrier function, low-Mw APS (< 10 kDa) may exert direct anti-inflammatory and anti-apoptotic effects. Advanced gut-targeted delivery systems are also discussed as strategies to enhance APS bioavailability and colonic targeting. Understanding these Mw-dependent mechanisms is critical for developing APS as a precise adjunct therapy to prevent cisplatin-induced AKI and improve patient outcomes.

## 1 Introduction

Cisplatin (CDDP) remains a cornerstone in the chemotherapy of various malignancies, including colorectal, lung, and ovarian cancers (Kelland, 2007; Dasari and Tchounwou, 2014). Despite its therapeutic efficacy, its clinical utility is frequently constrained by dose-limiting nephrotoxicity, with up to one-third of affected patients developing acute kidney injury (AKI; Miller et al., 2010; Pabla and Dong, 2008). Cisplatin-induced AKI

is a multifactorial process involving tubular cell injury, oxidative stress, and a robust inflammatory response. To date, no FDA-approved agents are available for its prevention or treatment (Ozkok and Edelstein, 2014; Zarbock et al., 2023).

In recent years, the gut microbiota has emerged as a critical modulator of drug efficacy and toxicity, including that of cisplatin (Ramezani and Raj, 2014; Meijers et al., 2019). This has led to the well-established concept of the gut–kidney axis, which posits that alterations in the composition and function of the intestinal microbiome exert direct and profound effects on kidney health and disease (Pluznick, 2020; Yang et al., 2020). Notably, cisplatin itself can damage the intestinal epithelium and disrupt the gut microenvironment, thereby inducing dysbiosis (Forslund et al., 2021; Maier et al., 2018).

Recent evidence indicates that cisplatin-induced AKI is closely linked to gut microbiota dysbiosis. Molecular-weight-optimized APS may provide renoprotection by restoring microbial balance and SCFA production. This review integrates clinical and literature data to propose a model of APS-mediated mitigation of cisplatin nephrotoxicity.

Fecal samples from 16 colorectal cancer patients were collected with informed consent. 16S rRNA gene sequencing was performed as described in previous publications (Wang et al., 2020; Mishima et al., 2017). Bioinformatics analysis assessed microbial diversity, taxonomic composition, and KEGG pathways.

These findings serve as the foundation for the central premise of this review: cisplatin creates a pathological gut microenvironment that exacerbates kidney injury, and targeting this dysbiosis represents a promising therapeutic strategy. Astragalus polysaccharides (APS), derived from the traditional Chinese herb *Astragalus membranaceus*, are recognized for their immunomodulatory, anti-inflammatory, and prebiotic properties (Liu et al., 2020; Zhao et al., 2023). Emerging evidence suggests that APS may protect against various forms of kidney injury, potentially through the restoration of gut microbial homeostasis (Liu W. et al., 2024; Song et al., 2022). However, APS is not a single entity but a complex mixture of polysaccharides with varying molecular weights (Mw). This structural diversity determines their biological fate and mechanisms of action. Low-Mw fractions may be directly absorbed, whereas high-Mw fractions function as prebiotics in the colon (Yu et al., 2018; Salamone et al., 2021).

The present review integrates clinical sequencing data on cisplatin-induced dysbiosis with current knowledge of APS pharmacology and advanced gut-targeted delivery systems. It proposes a clear and rational framework for using molecular-weight-optimized APS to precisely reverse the microbial and metabolic disruptions induced by cisplatin. This approach offers a novel, mechanism-based strategy for the prevention of chemotherapy-associated AKI.

## 2 The intestinal microbiota in cisplatin-triggered AKI: findings from 16S rRNA sequencing

The gut–kidney axis in cisplatin-induced AKI represents a dynamic, bidirectional interaction. Cisplatin not only damages the kidneys but also causes injury to the gastrointestinal tract, thereby establishing a self-perpetuating cycle that exacerbates systemic inflammation and renal injury (Ramezani and Raj, 2014; Gong et al., 2019). To comprehensively investigate the microbial alterations associated with this process, the authors performed 16S rRNA gene sequencing on fecal samples obtained from patients with colorectal cancer. The findings have direct and significant implications for the therapeutic application of APS.

### 2.1 Loss of microbial diversity and community structure perturbation

Given that high microbial diversity is a hallmark of a healthy gut ecosystem (Lozupone et al., 2012), the present analysis clearly demonstrates that cisplatin treatment significantly reduces this diversity.

**Figure 1:**
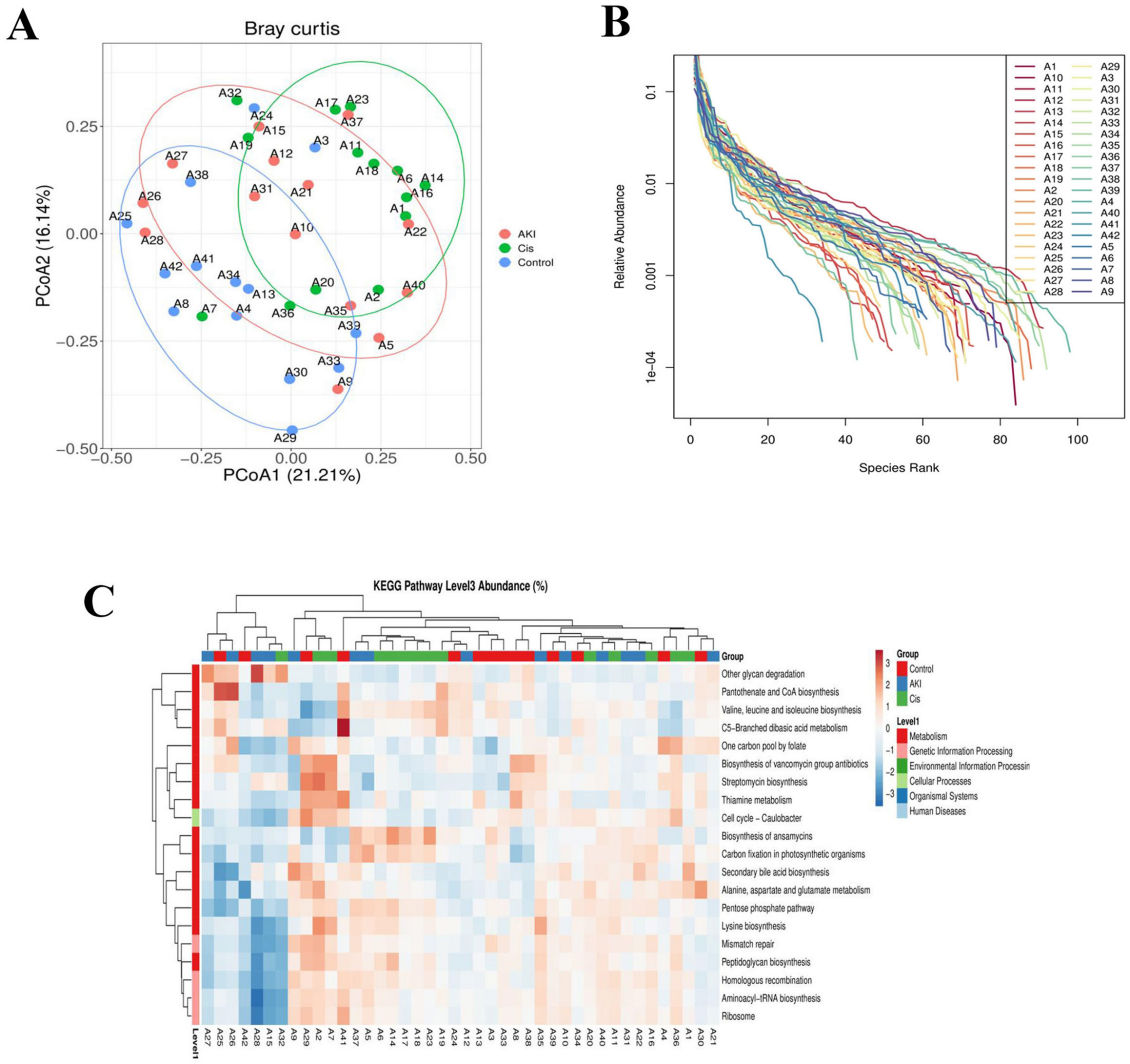
Diversity and community structure of gut microbiota in patients with colorectal cancer. **(A)** Principal Co-ordinate Analysis (PCoA) relying on Bray-Curtis distances reveals separate clustering of the Control (blue), Cisplatin-treated (green), and AKI (red) groups, suggesting notable disparities in the overall microbial community makeup. **(B)** Species richness curves contrasting the Cisplatin and Control groups, showing a decrease in microbial abundance and evenness among the Cisplatin-treated patients. **(C)** Heatmap depicting KEGG Pathway (Level 3) functional abundance, which demonstrates the different functional profiles of the three groups. The Cisplatin group exhibits changes in crucial metabolic pathways.

Figure 1A presents a principal coordinate analysis (PCoA) plot based on Bray–Curtis distances, illustrating the beta diversity of microbial communities. A clear and distinct separation is observed among the Control (blue), Cisplatin-treated (green), and AKI (red) groups. This clustering indicates that cisplatin chemotherapy induces a systemic and profound reorganization of the gut microbial community, consistent with prior reports that kidney injury leads to global structural alterations in the gut microbiota (Andrianova et al., 2020; Lee et al., 2020).

The species richness curve shown in Figure 1B further reveals a marked difference in microbial abundance between the Control and Cisplatin groups. The flatter curve for the Cisplatin group reflects a substantial decline in both species richness and evenness, indicating reduced ecosystem resilience. Consequently, the host becomes more susceptible to pathogen colonization and inflammation (Sommer et al., 2017).

These structural changes translate into functional consequences, as demonstrated by the KEGG pathway analysis in Figure 1C. The heatmap of Level 3 pathways shows a distinct functional profile in the Cisplatin group compared with the Control group, with notable alterations in metabolism-related pathways. These findings suggest that the functional capacity of the microbiota is also seriously compromised (Wang et al., 2020; Mishima et al., 2017).

### 2.2 Particular taxonomic alterations: reduction of beneficial microbes and accumulation of pathobionts

To identify which bacterial groups account for the observed community shifts, the taxonomic composition was analyzed in greater detail.

**Figure 2:**
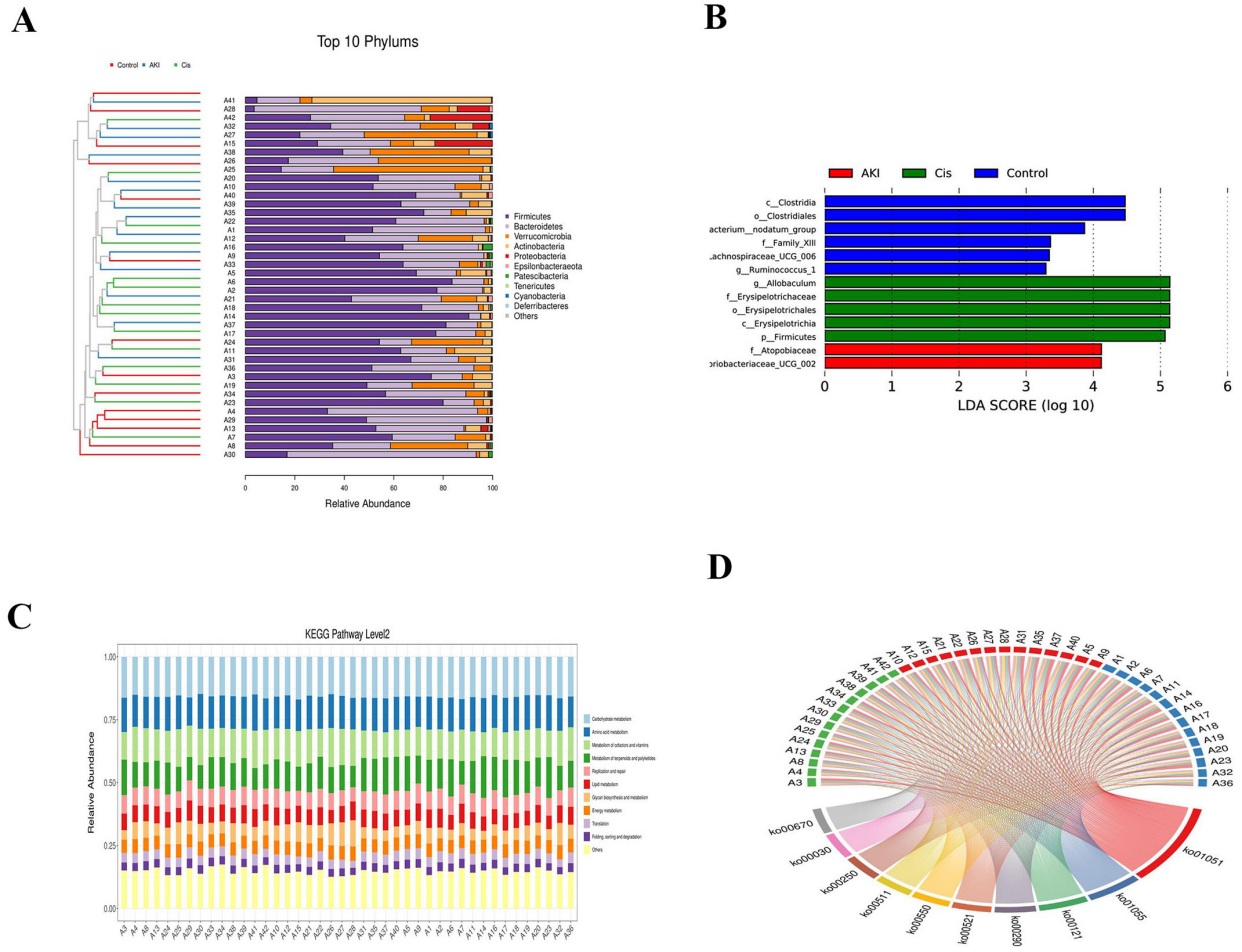
Influence of cisplatin on intestinal microbial composition and function. **(A)** Relative prevalence of the top 10 phyla among the Control, Cisplatin, and AKI sets, indicating alterations in the Firmicutes/Bacteroidetes proportion and a rise in Proteobacteria in the Cisplatin set. **(B)** Linear Discriminant Analysis (LDA) effect magnitude (LEfSe) presenting taxa that have notable differences in abundance among groups. The Cisplatin group is marked by a reduction of SCFA-producing bacteria such as *Faecalibacterium* and *Roseburia*. **(C)** KEGG Pathway (Level 2) functional abundance, emphasizing the down-regulation of crucial metabolic pathways in the Cisplatin group. **(D)** Chord graph depicting the intricate interactions among various groups and microbial functions, showing cisplatin-caused functional changes.

Figure 2A shows the relative abundances of the top 10 bacterial phyla among the Control, Cisplatin, and AKI groups. A key finding is the alteration in the Firmicutes/Bacteroidetes (F/B) ratio, a well-established indicator of gut dysbiosis (Vaziri et al., 2013). The decreased Firmicutes and increased Bacteroidetes and Proteobacteria in the Cisplatin group suggest a disruption of the commensal community structure.

To identify specific biomarkers associated with cisplatin treatment, we performed linear discriminant analysis effect size (LEfSe; Segata et al., 2011). Figure 2B presents the LDA scores, highlighting taxa with significant abundance differences between groups. Notably, the cisplatin-treated cohort exhibited a marked reduction in beneficial, short-chain fatty acid (SCFA)-producing bacteria, particularly from the genera *Faecalibacterium* and *Roseburia*. SCFAs, including butyrate, serve as the primary energy source for colonocytes and play an essential role in maintaining intestinal barrier integrity (Smith et al., 2013; Wang H. B. et al., 2012). Their depletion directly links gut dysbiosis to a leaky gut phenotype.

The LEfSe analysis, together with the chord diagram in Figure 2D, clearly demonstrates an increase in potentially harmful bacteria, especially those belonging to the phylum Proteobacteria, particularly the family *Enterobacteriaceae* (which includes *E. coli* and other opportunistic pathogens; Shin et al., 2015). Importantly, these bacteria possess urease and tryptophanase enzymes, which convert host-derived urea and dietary tryptophan into the uremic toxins indoxyl sulfate and p-cresyl sulfate (Gryp et al., 2017; Liu et al., 2018).

### 2.3 Functional outcomes: disruption of crucial metabolic pathways

To investigate the functional consequences of the observed taxonomic changes, KEGG pathway enrichment analysis was performed (Kanehisa and Goto, 2000).

**Figure 3:**
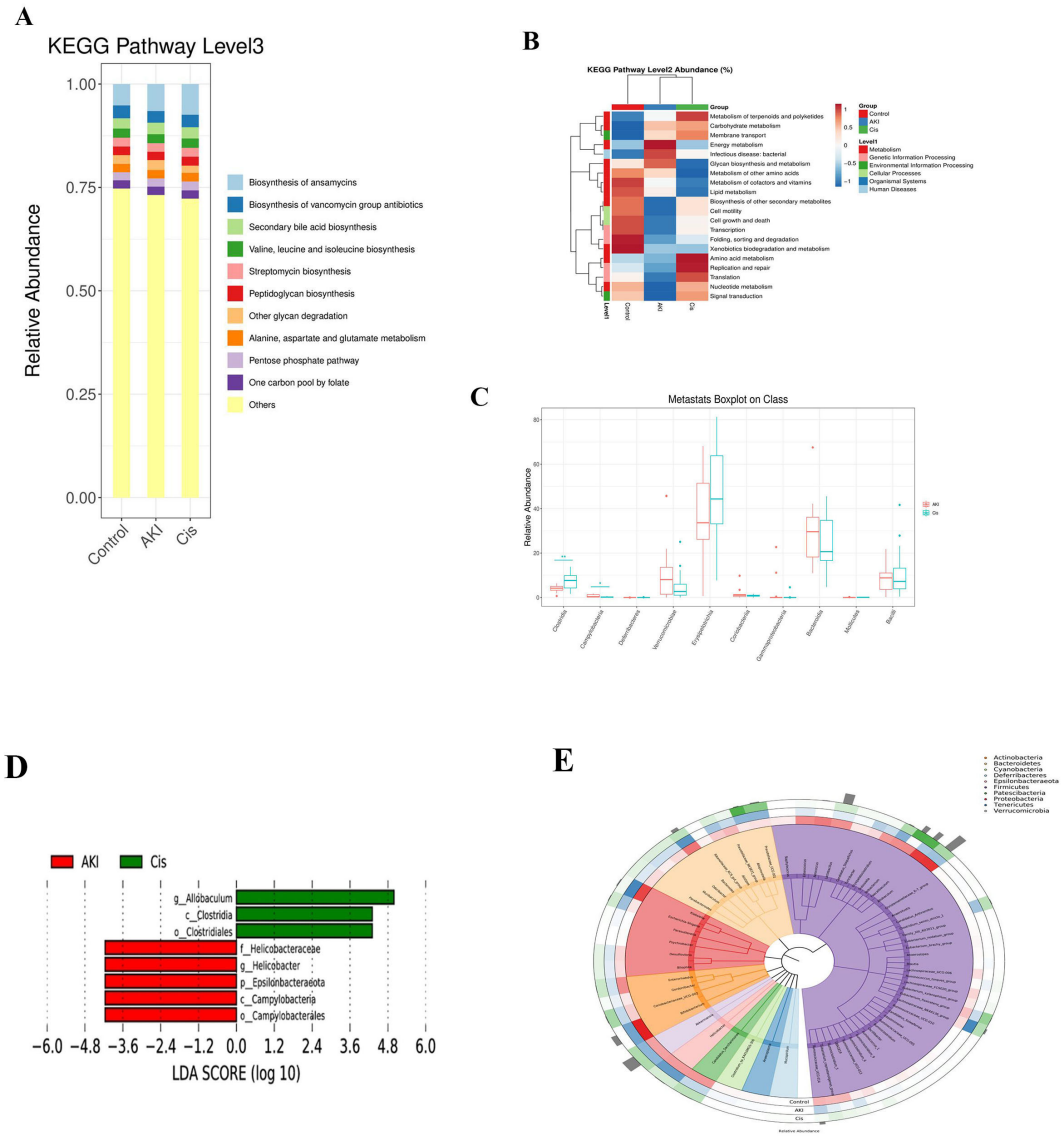
Functional examination of cisplatin-caused gut dysbiosis. **(A)** Dispersion of KEGG Pathway (Level 3) functional abundance among different groups. **(B)** Abundance of KEGG Pathway (Level 2), which further highlights the notable functional change induced by cisplatin. **(C)** Metastats assessment (box-shaped plots) indicating statistically notable differences in the abundance of particular functional characteristics between groups. **(D)** LDA scores for pinpointing notable taxonomic disparities between the Cisplatin and AKI groups at the species-level. **(E)** Classification tree showing the hierarchical framework and notable community disparities among the groups, validating the unique microbial profile of patients treated with cisplatin.

Figures 2C, 3A, B illustrate the alterations in KEGG pathways at Levels 2 and 3. A consistent finding is the marked downregulation of pathways essential for host health in the Cisplatin group. Specifically, we observed notable suppression of the following:

- **SCFA metabolism:** Given that SCFA metabolism is directly linked to the reduction of SCFA-producing bacteria such as *Faecalibacterium* and *Roseburia*, the decline in SCFAs likely impairs gut barrier integrity. This, in turn, promotes the translocation of bacterial products, including LPS, into the bloodstream—a well-recognized trigger of systemic inflammation in AKI (Smith et al., 2013; Andrade-Oliveira et al., 2015).
- **Amino acid metabolism:** The metabolism of histidine, tryptophan, and arginine was significantly downregulated. Disruption of tryptophan metabolism is known to disturb the balance of the kynurenine pathway, thereby impairing immune regulation and potentially contributing to patient fatigue and systemic symptoms (Cervenka et al., 2017; Platten et al., 2019).
- **Bile acid metabolism:** Alterations in bile acid metabolism, linked to reduced levels of bacteria such as *Bacteroides*, suggest a disturbance in enterohepatic circulation. This can impair lipid digestion and signaling, potentially leading to gastrointestinal discomfort and hepatic stress (Wahlström et al., 2016; Joyce and Gahan, 2016).

The Metastats analysis (Figure 3C), LDA scores for pinpointing notable taxonomic disparities between the Cisplatin and AKI groups at the species level (Figure 3D), and classification tree (Figure 3E) further substantiate these functional disparities (White et al., 2009), reinforcing the notion that cisplatin-induced dysbiosis is not merely taxonomic but also results in a significant loss of beneficial metabolic functions and the gain of potentially detrimental ones.

### 2.4 Summary: the cisplatin-induced dysbiosis signature

Based on the sequencing data presented, it is evident that cisplatin treatment induces a hostile gut environment characterized by reduced microbial diversity, depletion of SCFA-producing bacteria, and expansion of uremic toxin-generating microbes. Furthermore, this dysbiosis plays a central role in the inflammation and tissue damage associated with cisplatin-induced AKI, making it an optimal therapeutic target (Noel et al., 2014; Zhang et al., 2018). The main pathological outcomes of this dysbiosis are presented in Table 1. These findings provide a rationale for the application of molecular-weight-optimized APS to restore gut microbial homeostasis and mitigate cisplatin-induced AKI.

**Table 1:** Pathological consequences of gut dysbiosis in AKI.

Consequence	Mechanisms	Impact on AKI
Impaired SCFA production	A lower abundance of bacteria that generate SCFAs leads to a decrease in the concentrations of luminal SCFAs (Smith et al., 2013). SCFAs (particularly butyrate) preserve intestinal barrier integrity through upregulation of tight junctions (occludin, claudin-1, ZO-1) and mucus generation (Wang H. B. et al., 2012).	Impaired barrier function leads to an increase in permeability; the absence of anti-inflammatory SCFA effects intensifies systemic inflammation (Andrade-Oliveira et al., 2015; Macia et al., 2015).
Increased uremic toxin generation	The growth of *Enterobacteriaceae* leads to an increase in the production of indole (from tryptophan) and p-cresol (from tyrosine; Gryp et al., 2017). Absorbed and transformed into indoxyl sulfate and p-cresyl sulfate in the liver, these substances build up because of decreased renal clearance (Liu et al., 2018).	Toxins trigger inflammatory pathways, enhance oxidative stress, and directly harm tubular epithelial cells, intensifying kidney injury (Lim et al., 2021; Devlin et al., 2016).
Endotoxemia and systemic inflammation	A damaged intestinal barrier allows the movement of LPS and other bacterial substances into the bloodstream (Wang F. et al., 2012). LPS stimulates TLR4 on immune and kidney cells, initiating NF-κB-mediated inflammatory reactions (Maynard et al., 2012).	Elevated circulating endotoxin is associated with the severity of AKI and unfavorable outcomes (Onal et al., 2019).
Immune dysregulation	Gut microbiota influences systemic immunity; an imbalance in the gut microbiota changes the equilibrium between pro-inflammatory Th17 cells and anti-inflammatory Tregs (Song et al., 2022).	Triggers inflammation that sustains kidney damage; influences neutrophil recruitment, macrophage polarization, and dendritic cell function (Yang et al., 2021).

## 3 Astragalus polysaccharides: composition, molecular mass, and prebiotic capability

Given the well-established role of gut microbiota imbalance in cisplatin-induced AKI, strategies aimed at restoring microbial equilibrium are highly attractive. APS have emerged as a promising option due to their demonstrated ability to modulate the gut microbiota (Liu et al., 2020; Zhou et al., 2023). However, APS is not a homogeneous substance but rather a complex mixture of polysaccharides with varying molecular weights (Mw), and its biological activity is critically dependent on these structural features, particularly molecular mass (Yu et al., 2018).

### 3.1 Molecular weight-dependent physicochemical properties

The molecular weight of APS influences its solubility, viscosity, and, most importantly, its fate within the gastrointestinal tract (Salamone et al., 2021; Guo W. H. et al., 2024).

- **High molecular mass APS (>100 kDa):** These large polymers resist hydrolysis by human digestive enzymes and are not absorbed in the small intestine (Li M. et al., 2024). They remain largely intact and reach the colon, where they serve as a substrate (prebiotic) for the resident microbiota. Their high viscosity may also delay gastric emptying, potentially affecting nutrient absorption (Hu et al., 2025).
- **Low molecular weight APS (less than 10 kDa):** These smaller fragments exhibit lower viscosity and improved solubility (Sun et al., 2023). A small proportion may be directly absorbed via paracellular routes or through M cells in Peyer's patches, enabling potential direct interactions with the host's immune cells (Wu G. Z. et al., 2024). They are also more rapidly fermented by a broader range of gut bacteria, including various Firmicutes and Actinobacteria (Xue et al., 2025).
- **Medium molecular weight APS (10–100 kDa):** This fraction displays intermediate characteristics, sharing properties of both high and low Mw APS. It can be fermented by the gut microbiota while retaining some capacity for direct cellular interaction, representing a potential “sweet spot” for therapeutic activity (Xi et al., 2024; Deng et al., 2023).

### 3.2 Molecular weight as a determinant of prebiotic activity

The prebiotic function of APS—its ability to selectively promote the growth and activity of beneficial bacteria—is directly linked to its molecular weight (El Kaoutari et al., 2013; Cantu-Jungles and Hamaker, 2020).

High molecular-weight APS (>100 kDa) serve as prebiotics, fermented by Bacteroidetes with polysaccharide utilization loci, ensuring sustained SCFA production and continuous gut barrier support (Salyers et al., 1977; Martens et al., 2008). This gradual fermentation leads to a steady release of SCFAs, providing a long-term supply of beneficial metabolites to colonocytes (Harris et al., 2017). Such properties are particularly suitable for counteracting the persistent metabolic disturbances induced by cisplatin.

In contrast, low molecular weight APS are more readily accessible to a wider array of bacteria, including numerous Firmicutes and Actinobacteria such as *Bifidobacterium* (Song Q. Y. et al., 2024; Li et al., 2024). This results in a rapid burst of SCFA production, although the effect may be shorter-lived. By specifically enriching *Lactobacillus* and *Bifidobacterium*, low Mw APS can help restore populations of beneficial bacteria depleted by cisplatin (Zhang et al., 2024).

Given that the molecular weight profile of an APS formulation can be intentionally selected or engineered, a truly customized therapeutic effect is achievable: a blend of high and low Mw APS may provide both a rapid boost and sustained restoration of SCFA production, thereby directly and effectively addressing the metabolic deficiencies revealed by our sequencing data (Xi et al., 2024; Deng et al., 2023).

## 4 Mechanisms of APS-mediated kidney protection via microbiota modulation

Based on the gut dysbiosis pattern identified in our cisplatin-treated patients, the therapeutic mechanisms of APS can be viewed as a targeted strategy to reverse these specific pathological alterations. The molecular weight-dependent mechanisms are summarized in Table 2.

**Table 2:** Molecular weight-dependent mechanisms of APS in AKI.

Molecular weight	Primary mechanism	Key actions	Therapeutic application	References
High (>100 kDa)	Microbiota-dependent	Gradual fermentation; continuous SCFA generation; improved barrier performance; inhibition of uremic toxin-generating bacteria	AKI prevention in high-risk settings (prolonged protection desired)	Harris et al., 2017; Song et al., 2024; Zhou et al., 2023
Medium (10–100 kDa)	Balanced	Optimal fermentability featuring partial absorption; having both rapid and long-lasting dual effects; balanced short-chain fatty acid (SCFA) production and direct signaling regulation (hypothetical, inferred from blackberry polysaccharides and konjac glucomannans)	AKI treatment (optimal therapeutic window)	Xi et al., 2024; Deng et al., 2023
Low (< 10 kDa)	Direct cellular effects	Improved bioavailability; swift absorption; direct TLR4 regulation; anti-apoptosis; anti-oxidation; quick onset (hypothetical, inferred from other polysaccharides)	AKI acute therapy (timely intervention is crucial; might need repeated administration)	Wu G. Z. et al., 2024; Song Q. Y. et al., 2024; Li et al., 2024

### 4.1 Restoring SCFA production and gut barrier integrity

Our sequencing data revealed a marked reduction in SCFA-producing bacteria (*Faecalibacterium, Roseburia*) and downregulation of SCFA metabolic pathways. As a prebiotic, APS directly addresses this deficiency.

- **Mechanism:** High molecular weight APS are fermented by the remaining SCFA-producing bacteria, providing a substrate that supports their growth and the production of butyrate, acetate, and propionate (Waldecker et al., 2008; Arpaia et al., 2013).
- **Renal-protecting Effect:** Butyrate, in particular, enhances gut barrier function by upregulating tight junction proteins (e.g., occludin, claudin-1) and promoting mucus secretion (Smith et al., 2013; Kelly et al., 2015). By restoring SCFA generation, APS helps seal the leaky gut and prevents the translocation of LPS and other pro-inflammatory bacterial products into the bloodstream, which would otherwise exacerbate kidney inflammation (Andrade-Oliveira et al., 2015; Macia et al., 2015). This directly counteracts the endotoxemia-driven injury cycle.

### 4.2 Suppressing uremic toxin-producing bacteria

The LEfSe analysis clearly demonstrated an expansion of Proteobacteria, particularly Enterobacteriaceae, in patient samples. These bacteria are major producers of the uremic toxins indoxyl sulfate and p-cresyl sulfate (Gryp et al., 2017; Liu et al., 2018). In AKI, impaired renal clearance leads to the accumulation of these toxins, which directly contribute to tubular cell damage and fibrosis (Lim et al., 2021; Devlin et al., 2016).

- **Mechanism:** APS can inhibit the growth of these pathobionts, primarily indirectly, by promoting the proliferation of beneficial bacteria that compete for resources and ecological niches (Kamada et al., 2013; Buffie and Pamer, 2013). Some evidence also suggests that fermentation products of APS create a microenvironment less favorable to these harmful agents (Dong et al., 2019).

By suppressing toxin-producing bacteria, APS reduces the levels of indoxyl sulfate and p-cresyl sulfate, thereby eliminating a direct trigger of tubular epithelial cell stress and inflammation, and exerting a clear renoprotective effect (Pluznick et al., 2013).

### 4.3 Modulating systemic inflammation and immune homeostasis

Cisplatin-induced AKI is characterized by a robust inflammatory response (Ozkok and Edelstein, 2014; Linkermann et al., 2014); therefore, it is not surprising that our KEGG analysis revealed disruptions in immune-related pathways. Furthermore, the depletion of SCFAs—potent immunomodulators—directly exacerbates the pro-inflammatory state.

- **SCFA-GPR41/43 signaling pathway:** SCFAs generated from APS fermentation activate the G-protein-coupled receptors GPR41 and GPR43 on immune cells and renal epithelial cells (Brown et al., 2003; Maslowski et al., 2009). This activation promotes the differentiation of anti-inflammatory regulatory T cells (Tregs) and suppresses the production of pro-inflammatory cytokines such as TNF-α and IL-1β (Furusawa et al., 2013; Thangaraju et al., 2009).
- **TLR4/NF-κB pathway regulation:** Low molecular weight APS may directly interact with Toll-like receptor 4 (TLR4) on immune cells, potentially acting as a competitive inhibitor of LPS and suppressing the TLR4/NF-κB pro-inflammatory pathway (Li R. Y. et al., 2024; Ke et al., 2017). This dual mechanism, combining direct and microbiota-mediated effects, positions APS as a potent modulator of the systemic inflammation observed in cisplatin-treated patients (Al-Harbi et al., 2018).

## 5 Signaling pathways mediating APS renal protection

The renoprotective effects of APS are mediated through multiple interconnected signaling pathways, as summarized in Table 3.

**Table 3:** Signaling pathways mediating APS renal protection.

Pathway	Mechanism of activation	Protective effects	Molecular weight dependence
SCFA-GPR41/43	SCFAs from APS fermentation activate GPR41/43 on renal and immune cells (Brown et al., 2003; Maslowski et al., 2009)	Inhibits NF-κB, restrains TNF-α/IL-1β/IL-6; facilitates Treg differentiation through HDAC inhibition; boosts tight junction expression (Wang H. B. et al., 2012; Thangaraju et al., 2009; Le Poul et al., 2003; Carretta et al., 2013)	High molecular weight: continuous activation; Low molecular weight: short-term effects (Deng et al., 2023)
TLR4/NF-κB	Competitive binding of TLR4 by APS fractions having α-1,6-linked galactose or arabinogalactan side-chains (Liu et al., 2020; Ke et al., 2017); Inhibition of HDAC mediated by SCFA also inhibits NF-κB (Singh et al., 2014)	Decreases NF-κB p65 nuclear migration; inhibits pro-inflammatory cytokine generation (Dong et al., 2019)	Low Molecular Weight: direct binding to TLR4; High Molecular Weight: mediated by SCFA (Andrade-Oliveira et al., 2015)
Nrf2/HO-1	Direct modification of KEAP1 by low molecular weight APS (Itoh et al., 1999); Nrf2 activation mediated by SCFA through HDAC inhibition (Wang et al., 2017)	Elevates HO-1, NQO1, GST expression; lessens oxidative stress and lipid peroxidation; maintains mitochondrial function (Braga et al., 2017; Hu et al., 2023)	Low molecular weight: direct and swift activation; High molecular weight: short-chain fatty acid (SCFA)-mediated (Wang et al., 2017)
PI3K/Akt	Direct interactions with receptors (TLR4) or induction of growth factors (Ke et al., 2017); Signaling of SCFA-GPR also plays a role (Zhou et al., 2021)	Phosphorylates and renders BAD and caspase-9 inactive; raises the Bcl-2/Bax ratio; lessens apoptosis (Zhou et al., 2021; He Y. X. et al., 2024)	Low molecular weight: direct activation; High molecular weight: SCFA-mediated (Jiang et al., 2024)
NLRP3 Inflammasome	SCFA-induced inhibition through GPR109A signaling and HDAC suppression (Singh et al., 2014; Macia et al., 2015); direct impacts on mitochondrial ROS by low-molecular-weight APS (Pan et al., 2022; Zhong et al., 2024)	Lowers IL-1β and IL-18 generation; weakens caspase-1 activation; restricts pyroptosis (Braga et al., 2017; Foresto-Neto et al., 2018; Chen et al., 2024; Ruan et al., 2023)	Low molecular weight: direct; High molecular weight: short-chain fatty acid (SCFA)-mediated

### 5.1 SCFA-GPR41/43 signaling axis

SCFAs generated through APS fermentation activate the G-protein-coupled receptors GPR41 (FFAR3) and GPR43 (FFAR2), which are expressed on kidney cells, immune cells, and enteroendocrine cells (Brown et al., 2003). In ischemia-reperfusion injury (IRI) models, APS treatment enhanced renal GPR43 expression and attenuated injury, effects that were abolished by GPR43 antagonists or in GPR43-deficient mice (Maslowski et al., 2009).

Activation of GPR41/43 by APS-derived SCFAs exerts multiple protective effects: (1) inhibition of the NF-κB signaling pathway and reduction of pro-inflammatory cytokines (TNF-α, IL-1β, and IL-6; Le Poul et al., 2003); (2) facilitation of regulatory T cell (Treg) differentiation via histone deacetylase (HDAC) inhibition and epigenetic regulation of Foxp3 (Thangaraju et al., 2009); (3) improvement of intestinal barrier function through upregulation of tight junction proteins (Wang H. B. et al., 2012); and (4) regulation of neutrophil recruitment and function (Carretta et al., 2013).

The extent of GPR41/43 activation depends on the SCFA concentrations achieved, which vary with APS molecular weight and fermentation kinetics. High molecular weight APS, which support sustained SCFA production, may maintain long-term receptor activation based on fermentation studies (Deng et al., 2023); low molecular weight fractions may produce more transient effects, as hypothesized from their faster absorption and lower fermentation rates.

### 5.2 TLR4/NF-κB pathway modulation

APS modulates TLR4/NF-κB signaling through multiple mechanisms that depend on molecular weight and structural characteristics (Zhao et al., 2023).

- **Competitive TLR4 Binding:** APS fractions with specific branching structures (α-1,6-linked galactose or arabinogalactan side chains) bind TLR4 with affinities similar to LPS, potentially competing with endotoxin for receptor occupancy (Liu et al., 2020). This competitive binding could reduce LPS-induced inflammatory reactions without initiating complete activation (Ke et al., 2017).
- **Suppression of NF-κB nuclear movement:** APS treatment reduces NF-κB p65 nuclear translocation in kidney epithelial cells and immune cells, thereby inhibiting the transcription of pro-inflammatory genes (Dong et al., 2019). This effect is achieved through inhibition of IκBα phosphorylation and degradation (Sun et al., 2013).
- **Downstream cytokine inhibition:** APS curtails the production of TNF-α, IL-1β, IL-6, and other NF-κB-dependent cytokines in AKI models, mitigating inflammatory damage (Andrade-Oliveira et al., 2015; Al-Harbi et al., 2018). These effects are partly microbiota-dependent, as SCFAs also suppress NF-κB via HDAC inhibition (Singh et al., 2014).
- **Regarding molecular weight dependence:** low molecular weight APS with appropriate branching may directly engage TLR4 (hypothetical, inferred from structural analogy with other polysaccharides), whereas high molecular weight fractions rely on SCFA-mediated effects supported by experimental studies (Singh et al., 2014; Andrade-Oliveira et al., 2015).

### 5.3 Nrf2/HO-1 antioxidant pathway activation

APS activates the Nrf2 antioxidant pathway, increasing the expression of cytoprotective enzymes such as heme oxygenase-1 (HO-1), NAD(P)H quinone oxidoreductase 1 (NQO1), and glutathione S-transferases (Braga et al., 2017). Nrf2 activation occurs via both direct and indirect mechanisms (Itoh et al., 2004).

- **Direct activation of Nrf2:** Low-molecular-weight APS might directly alter KEAP1 cysteine residues, enabling Nrf2 nuclear translocation (hypothetical, inferred from studies on other polysaccharides; not directly tested for APS; Itoh et al., 1999); high-mass APS activate Nrf2 via SCFA-mediated mechanisms supported by experimental evidence (Wang et al., 2017). This direct effect provides rapid antioxidant defense independent of the microbiota (Li et al., 2008).
- **Activation of Nrf2 Mediated by SCFA:** Butyrate and other short-chain fatty acids (SCFAs) trigger Nrf2 activation via HDAC inhibition and regulation of KEAP1 expression (Wang et al., 2017). High molecular weight APS thus promote Nrf2 activation through microbiota-dependent mechanisms (Yin et al., 2026).
- **Implications for AKI:** Nrf2 activation reduces oxidative stress in AKI models, decreasing lipid peroxidation (MDA), preserving mitochondrial function, and attenuating tubular cell death (2023). APS therapy enhances renal HO-1 expression and reduces injury in IRI and nephrotoxin models (Nakade et al., 2018; Shimasaki et al., 2019).

### 5.4 PI3K/Akt survival pathway

APS activates the PI3K/Akt signaling pathway in kidney cells, promoting cell survival and inhibiting apoptosis (Xu et al., 2021). Akt phosphorylation increases following APS treatment in cultured tubular epithelial cells exposed to hypoxia or toxins (Chen et al., 2021).

- **Activation mechanisms:** PI3K/Akt activation by APS may involve direct interactions with receptors (e.g., TLR4 or other pattern-recognition receptors) or indirect effects via induction of growth factors (Ke et al., 2017). SCFAs may also contribute to Akt activation through GPR-mediated signaling (Zhou et al., 2021).
- **Anti-cell-death effects:** Akt phosphorylates and inactivates pro-apoptotic proteins (BAD, caspase-9) while enhancing the expression of anti-apoptotic Bcl-2 family members (He Y. X. et al., 2024). APS treatment increases the Bcl-2/Bax ratio and reduces caspase-3 activation in AKI models (Zhou et al., 2021; He Y. X. et al., 2024).
- **Dependence on molecular weight:** Low molecular weight APS may activate PI3K/Akt more efficiently through direct cell interactions (hypothetical, inferred from other polysaccharides), whereas high molecular weight APS exert effects largely mediated by SCFAs, as supported by *in vitro* and *in vivo* data (Zhou et al., 2021; Jiang et al., 2024).

### 5.5 NLRP3 inflammasome regulation

The NLRP3 inflammasome contributes to AKI pathogenesis by producing IL-1β and IL-18 and inducing pyroptosis (Braga et al., 2017). The NLRP3 inflammasome contributes to AKI pathogenesis by producing IL-1β and IL-18 and inducing pyroptosis (Foresto-Neto et al., 2018).

- **SCFA-induced suppression:** Butyrate suppresses NLRP3 activation via GPR109A signaling and HDAC inhibition, leading to reduced IL-1β production (Singh et al., 2014; Macia et al., 2015). Thus, APS-generated SCFAs inhibit inflammasome activation.
- **Direct impacts:** Low-molecular-weight APS might directly regulate NLRP3 via mitochondrial and ROS effects (hypothetical, inferred from mechanistic studies on other polysaccharides; not directly tested for APS; Pan et al., 2022); high-molecular-weight APS inhibit NLRP3 through SCFA-mediated pathways supported by experimental studies (Singh et al., 2014; Macia et al., 2015). APS treatment reduces mitochondrial ROS and suppresses NLRP3 oligomerization in experimental settings (Zhong et al., 2024).
- **Outcomes for AKI:** NLRP3 inhibition by APS alleviates renal inflammation and tubular damage in IRI and nephrotoxin models (Chen et al., 2024; Ruan et al., 2023). These effects are associated with decreased IL-1β and IL-18 levels and reduced caspase-1 activation.

## 6 Gut-targeted delivery systems for APS

Conventional oral APS formulations face considerable challenges, including degradation by gastric acid and digestive enzymes, suboptimal absorption in the small intestine, non-specific distribution, and difficulties in achieving targeted colonic release (Guo W. H. et al., 2024; Salamone et al., 2021). To address these issues, various advanced delivery systems have been developed.

### 6.1 ph-responsive colon-targeted delivery systems

The pH gradient along the gastrointestinal tract provides a basis for colon-targeted delivery: gastric pH ranges from 1.5 to 3.5, small intestinal pH from 6.0 to 7.0, and colonic pH from 5.5 to 7.0 (slightly lower due to SCFA production; Guo et al., 2023).

- **pH-responsive polymer coatings:** APS formulations can be coated with pH-sensitive polymers (e.g., Eudragit S100) to ensure colonic release and microbial fermentation. This approach is supported by APS-specific studies demonstrating enhanced SCFA-mediated effects *in vivo* (Song et al., 2024; Zhou et al., 2023). This system is particularly suitable for delivering high molecular weight APS, ensuring they reach the colon intact for fermentation (Chuang et al., 2024).
- **pH-responsive hydrogels:** Hydrogel microspheres based on chitosan-alginate exhibit pH responsiveness: they shrink in gastric acid to protect APS and swell at colonic pH to release the drug (Li X. M. et al., 2024). Studies have shown that APS-encapsulated chitosan-alginate hydrogels significantly increase local APS concentrations in the colon and improve therapeutic outcomes in colitis models (Tang et al., 2025).

### 6.2 Nanocarrier delivery systems

Chitosan, a positively charged polysaccharide with mucoadhesive and absorption-enhancing properties, can be used to prepare APS-loaded nanoparticles (100–300 nm) via ionic gelation (Fan et al., 2022). Chitosan nanoparticles protect APS from degradation, improve intestinal epithelial retention and absorption, and facilitate lymphatic transport through M cells in Peyer's patches (Liu et al., 2023). Research indicates that oral administration of APS-loaded chitosan nanoparticles markedly increases APS distribution in colonic tissue and enhances gut microbiota regulation (Song et al., 2024).

- **Liposomes:** Liposomes, lipid-based bilayer vesicles, can encapsulate hydrophilic APS. Surface-modified liposomes (e.g., PEGylated liposomes) prolong circulation time, whereas ligand-modified liposomes (e.g., folate or lectin conjugation) enable active targeting (Zhou et al., 2023). Oral liposomes protect APS from gastrointestinal breakdown and are taken up via M cells and the intestinal lymphatic pathway (Liang et al., 2024).
- **Solid lipid nanoparticles:** Solid lipid nanoparticles combine the advantages of liposomes and polymeric nanoparticles, offering high drug loading capacity, favorable stability, and controlled-release characteristics (Liu S. Y. et al., 2024). APS-loaded solid lipid nanoparticles have shown improved intestinal absorption and bioavailability (Xu et al., 2024).

### 6.3 Probiotic-based live biotherapeutic delivery systems

- **Probiotics acting as living carriers:** Certain probiotics (e.g., *Lactobacillus, Bifidobacterium, Escherichia coli* Nissle 1917) possess intrinsic intestinal colonization capacity and probiotic benefits, serving as living carriers for APS delivery (Song et al., 2025). APS can be attached to probiotic surfaces via non-covalent bonding or surface display techniques, enabling targeted intestinal delivery and sustained release (Wu G. et al., 2024).
- **Probiotic-polysaccharide combinations:** Studies indicate that fermentation of APS by *Lactobacillus* generates metabolites with enhanced bioactivity (He et al., 2024). Combining APS with probiotics (e.g., *Lactobacillus, Bifidobacterium*) synergistically boosts SCFA production and microbiota modulation (Rydzewska-Rosolowska et al., 2020).
- **Genetically modified probiotics:** Genetic engineering techniques can be used to modify probiotics to express specific polysaccharide-degrading enzymes or therapeutic proteins (Chen et al., 2025). For example, genetically engineered *E. coli* Nissle 1917 expressing fucose transporters and metabolic enzymes enables targeted delivery and release of therapeutic molecules (Venegas et al., 2019).

### 6.4 Hydrogel delivery systems

- ***In situ* forming hydrogels:** Thermosensitive or pH-responsive hydrogels can form gels in situ following oral administration, thereby extending intestinal retention of APS (Emal et al., 2017). Chitosan-β-glycerophosphate thermosensitive hydrogels remain liquid at room temperature and gel at body temperature, facilitating sustained APS release (Jang et al., 2009).
- **Microneedle hydrogels:** Recently, polysaccharide-based hydrogel microneedle patches have been developed for transmucosal delivery. Although primarily intended for local applications, they offer novel perspectives for intestinal targeted delivery (Ubeda and Pamer, 2012).

### 6.5 Optimized delivery strategies for different molecular weight APS

Based on the properties of APS that are related to molecular weight, optimized delivery approaches can be devised:

APS Type Delivery System Selection Design Rationale Expected Outcome:

- High Mw (>100 kDa) pH-responsive colon-targeted coating, prebiotic-probiotic complexes Protection through upper GI tract, ensuring intact colonic delivery Slow colonic fermentation, sustained SCFA production.
- Medium Mw (10–100 kDa) Nanoparticles, liposomes, hydrogels Controlled release combining colonic delivery and partial absorption Rapid SCFA production with partial direct effects.
- Low Mw (< 10 kDa) Nanoparticles, liposomes, probiotic surface display Enhanced absorption and bioavailability, immune cell targeting Rapid systemic anti-inflammatory and anti-apoptotic effects.

Based on the molecular weight-related properties of APS, optimized delivery strategies can be designed, as summarized in Table 4.

**Table 4:** Optimized delivery strategies for different molecular weight APS.

APS type	Delivery system selection	Design rationale	Expected outcome
High Mw (>100 kDa)	pH-responsive coatings; prebiotic-probiotic complexes	Protection through upper GI tract, ensuring colonic delivery	Slow fermentation, sustained SCFA production
Medium Mw (10–100 kDa)	Nanoparticles,liposomes, hydrogels	Controlled release combining colonic delivery and partial absorption	Rapid SCFA production with direct effects
Low Mw (< 10 kDa)	Nanoparticles, liposomes, probiotic surface display	Enhanced absorption and bioavailability; immune cell targeting	Rapid systemic anti-inflammatory and anti-apoptotic effects

## 7 APS with optimized molecular weight for cisplatin-caused AKI: an integrated model

Integrating our clinical sequencing data with the molecular weight-dependent pharmacology of APS, we propose a targeted therapeutic model for cisplatin-induced AKI (Guo et al., 2024; Tang et al., 2023).

- **Focusing on the cisplatin-induced dysbiosis signature:** The primary goals of APS intervention should be to (1) increase the abundance of SCFA-producing bacteria (*Faecalibacterium, Roseburia*), (2) reduce the population of uremic toxin-producing bacteria (Enterobacteriaceae), and (3) restore SCFA and amino acid metabolic pathways (He et al., 2024; Rydzewska-Rosolowska et al., 2020).
- **Function of high molecular weight APS (>100 kDa):** These fractions serve as the sustained-release backbone of the treatment. Their slow fermentation leads to stable, long-lasting SCFA release in the distal colon, thereby maintaining gut barrier integrity over time and continuously activating anti-inflammatory pathways (Harris et al., 2017; Venegas et al., 2019). This makes them well-suited to counteract cisplatin-induced dysbiosis and metabolic disturbances.
- **Function of Low molecular-weight APS (less than 10 kDa):** These act as a rapid-response component. They can be quickly fermented by residual beneficial bacteria to provide an immediate boost in SCFAs—a hypothesized effect based on studies of other polysaccharides, such as those from *Artemisia argyi* and *Undaria pinnatifida* (Song Q. Y. et al., 2024; Li et al., 2024). Moreover, their potential for direct uptake may exert rapid systemic anti-inflammatory and anti-apoptotic effects on renal tubular cells, offering a first line of defense immediately following cisplatin administration (Wu G. Z. et al., 2024; Xu et al., 2024).
- **Function of medium molecular weight APS (10–100 kDa):** This fraction may offer a balanced approach, being accessible to a broader range of bacteria than high Mw APS while providing more sustained effects than low Mw APS (Xi et al., 2024; Deng et al., 2023). It may be particularly effective in promoting the growth of *Lactobacillus* and *Bifidobacterium* species—a hypothesis inferred from studies of other polysaccharides, such as blackberry polysaccharides and konjac glucomannans (Xi et al., 2024; Deng et al., 2023; Song et al., 2025).

A suggested Mw-optimized APS formulation for cisplatin-induced AKI would therefore be a combination: a substantial proportion of high Mw APS to ensure sustained distal gut SCFA production, together with low Mw APS to provide rapid systemic anti-inflammatory effects (Liang et al., 2024; Liu S. Y. et al., 2024).

## 8 Clinical translation and future perspectives

### 8.1 Current clinical evidence

Clinical studies of *Astragalus* in kidney disorders provide proof-of-concept for the therapeutic potential of APS, although direct evidence in AKI remains limited (Liu M. et al., 2024).

- **CKD research:** A multicenter randomized controlled trial in patients with type 2 diabetes and CKD stages 2–3 showed that add-on *Astragalus* granules (7.5 g/day for 48 weeks) significantly slowed the decline in eGFR compared with standard therapy alone (difference 4.6 mL/min/1.73 m^2^ per year; Chan et al., 2024). A large-scale cohort study in Taiwan reported that Chinese herbal medicine containing *Astragalus* (ASRD formula) reduced the risk of end-stage renal disease (aHR 0.83) and all-cause mortality (aHR 0.78) in patients with advanced CKD, without increasing the risk of hyperkalemia (Chen et al., 2022).
- **AKI research:** Clinical trials specifically investigating APS in AKI are lacking. A randomized trial of probiotics and prebiotics in septic AKI has been conducted (NCT03877081), but results have not yet been published (Chávez-Íñiguez et al., 2023). A retrospective analysis in cirrhotic patients indicated that rifaximin—a non-absorbable antibiotic targeting the gut microbiota—reduced the incidence of AKI, supporting the therapeutic potential of microbiota modulation (Dong et al., 2016).
- **Safety information:** *Astragalus* and APS have demonstrated favorable safety profiles in clinical studies, with adverse event rates similar to placebo and no increased risk of hyperkalemia or other serious complications (Chan et al., 2024; Chen et al., 2022). However, long-term safety data and studies specifically in AKI populations are needed (Chan et al., 2024).

### 8.2 Biomarker-guided patient selection

Biomarkers may enable individualized APS therapy in AKI by identifying patients most likely to benefit (Dasari and Tchounwou, 2014).

- **Microbiota-related biomarkers:** Baseline gut microbiota composition (diversity metrics, abundance of SCFA producers, prevalence of toxin producers) may predict response to APS (Wu G. et al., 2024). Patients with low baseline levels of SCFA-producing bacteria may derive the greatest benefit from APS supplementation (Bennett and Devarajan, 2011).
- **Metabolite-based biomarkers:** Baseline SCFA levels and uremic toxin concentrations could identify patients with microbiota dysfunction suitable for APS intervention (Yu et al., 2024). Monitoring changes in these metabolites may guide dose optimization and treatment duration (He et al., 2024).
- **Genetic biomarkers:** Variations in SCFA receptors (GPR41, GPR43) or TLR4 may influence APS response and enable genotype-directed therapy (Chen et al., 2025).

### 8.3 Future directions

Future research directions include: (1) preclinical validation of Mw-specific APS fractions in cisplatin-induced AKI models; (2) mechanistic studies using gnotobiotic mice to identify key microbial mediators; and (3) clinical translation incorporating gut microbiota biomarkers to optimize APS intervention (Chan et al., 2024; Chen et al., 2022). Given the comprehensive exploration of the therapeutic potential of molecular-weight-optimized APS in regulating the gut–kidney axis, it is now feasible to establish a novel precision medicine model to protect patients from the nephrotoxic side effects of life-saving chemotherapy (Ronco et al., 2019; Hoste et al., 2015).

## 9 Conclusions

Cisplatin-induced AKI remains a major clinical challenge with limited preventive options. Our analysis highlights the critical role of gut microbiota dysbiosis in the pathogenesis of cisplatin nephrotoxicity, characterized by reduced SCFA-producing bacteria, increased uremic toxin-generating microbes, and disturbances in key metabolic pathways.

Molecular-weight-optimized APS offer a promising strategy to modulate the gut–kidney axis and mitigate AKI. High molecular weight fractions support sustained SCFA production, while low molecular weight fractions may exert rapid anti-inflammatory effects. Advanced gut-targeted delivery systems enhance APS bioavailability and enable colon-specific release, thereby optimizing their therapeutic potential.

Overall, APS represent a tunable therapeutic system that, through modulation of the gut microbiota and related metabolic and immune pathways, can provide renoprotection and improve outcomes in patients undergoing cisplatin chemotherapy. This review underscores the potential of Mw-optimized APS as a precision adjunct therapy against chemotherapy-induced nephrotoxicity.
